# Obesity, sarcopenia, sarcopenic obesity and reduced mobility in Brazilian older people aged 80 years and over

**DOI:** 10.1590/S1679-45082017AO4058

**Published:** 2017

**Authors:** Vanessa Ribeiro dos Santos, Igor Conterato Gomes, Denise Rodrigues Bueno, Diego Giulliano Destro Christofaro, Ismael Forte Freitas, Luis Alberto Gobbo

**Affiliations:** 1 Programa de Pós-Graduação em Ciências da Motricidade, Faculdade de Ciências e Tecnologia, Universidade Estadual Paulista, Presidente Prudente, SP, Brazil; 2 Faculdade Maurício de Nassau, Natal, RN, Brazil; 3 Universidade Tecnológica Federal do Paraná, Apucarana, PR, Brazil

**Keywords:** Aging, Body composition, Gait, Densitometry, Aged, 80 and over, Aged, Sarcopenia, Obesity, Mobility limitation, Envelhecimento, Composição corporal, Marcha, Densitometria, Idoso de 80 anos ou mais, Idoso, Sarcopenia, Obesidade, Limitação da mobilidade

## Abstract

**Objective:**

To analyze which abnormalities in body composition (obesity, sarcopenia or sarcopenic obesity) are related to reduced mobility in older people aged 80 years and older.

**Methods:**

The sample included 116 subjects aged 80 years and older. The body composition was measured using dual-energy X-ray absorptiometry (DXA) and mobility was assessed by motor tests. The χ^2^ test was used to analyze the proportion of older people with sarcopenia, obesity and sarcopenic obesity based on sex as well as to indicate an association between obesity, sarcopenia, sarcopenic obesity and mobility. Binary logistic regression, adjusted for the variables (sex and osteoarticular diseases), was used to express the magnitude of these associations. One-way analysis of variance was used to compare the mobility of four groups (Normal, Obesity, Sarcopenia and Sarcopenic Obesity).

**Results:**

The Sarcopenia Group had lower performance in the lower limbs strength test and in sum of two tests compared with Obesity and Normal Groups. Older people with sarcopenia had higher chance of reduced mobility (OR: 3.44; 95%CI: 1.12-10.52).

**Conclusion:**

Older people aged 80 years and older with sarcopenia have more chance for reduction in mobility.

## RESUMO

**Objetivo:**

Analisar quais agravos na composição corporal (obesidade, sarcopenia ou obesidade sarcopênica) estão relacionados à redução da mobilidade em idosos com 80 anos ou mais.

**Métodos:**

A amostra foi constituída por 116 sujeitos com idade igual ou superior a 80 anos. A composição corporal foi mensurada pela técnica de absorciometria de raios X de dupla energia (DXA), e a mobilidade foi avaliada por testes motores. O teste χ^2^foi utilizado para analisar a proporção de idosos com sarcopenia, obesidade e obesidade sarcopênica, de acordo com o sexo, bem como para indicar a associação entre obesidade, sarcopenia, obesidade sarcopênica e mobilidade. A análise de regressão logística binária, ajustada pelas variáveis (sexo e doenças osteoarticulares), foi utilizada para expressar a magnitude das associações. A análise de variância foi usada para comparar a mobilidade entre os quatro grupos (Normal, Obesidade, Sarcopenia e Obesidade Sarcopênica).

**Resultados:**

O Grupo Sarcopenia apresentou menor desempenho no teste de força de membros inferiores e na soma dos dois testes, comparado aos Grupos Obesidade e Normal. Idosos com sarcopenia apresentaram maior chance de redução da mobilidade (OR: 3,44; IC95%: 1,12-10,52).

**Conclusão:**

Idosos com idade igual ou superior a 80 anos com sarcopenia têm mais chance de redução da mobilidade.

## INTRODUCTION

Advanced age is a risk factor for reduced mobility, and when age is associated with changes in body composition, such as the relative increase in body fat in relation to lean mass,^(^ ^1^ ^)^ the deleterious effects may be potentiated.^(^ ^2^ ^)^ A marked decrease in muscle mass that occurs with aging, associated with low muscle strength or low physical performance, is defined as sarcopenia.^(^ ^3^ ^)^ When this condition coexists with excess of body fat, it is defined as sarcopenic obesity.^(^ ^4^ ^)^

Reduction in mobility can be considered as an indicator of health because it affects quality of life in aging process by causing difficult in some daily life activities and the use of public transport, therefore, directly affecting the independence of older people. Another serious consequences of the development of physical impairment are the increased need for hospitalization and the use of the health services,^(^ ^5^ ^)^ which result in a negative impact on the public health system.

Evidence indicates that sarcopenia,^(^ ^6^ ^,^ ^7^ ^)^ obesity^(^ ^8^ ^,^ ^9^ ^)^ and sarcopenic obesity^(^ ^10^ ^,^ ^11^ ^)^ are predictors of reduced mobility, however, it has become necessary to investigate which of these conditions is more related to reduced mobility in older people aged 80 years or older, particularly because subjects belonging to this age group are more predisposed to disability.^(^ ^12^ ^)^

## OBJECTIVE

To determine which abnormalies in body composition (obesity, sarcopenia or sarcopenic obesity) are related to reduced mobility in older people aged 80 years and older.

## METHODS

This was a cross-sectional study with a non-randomized convenience sample conducted between October 2009 and May 2010 in the city of Presidente Prudente (SP) Brazil, with 210,000 habitants approximately located in the Southeastern Region of Brazil.^(^ ^13^ ^)^

Older people, aged 80 years and older of both sexes were invited to participate in the study. The Presidente Prudente municipalilty health department provided the names, addresses and telephone numbers of individuals who used the public health service of the city. The invitation was made via telephone and, in addition, the research was also disclosed in the local media. A total of 135 subjects responded the invitation. We excluded those who were unable to walk, bedridden, residents of rural areas, institutionalized, with pacemakers and had incomplete data in the database. The final sample consisted of 116 individuals.

Objectives and methods used for data collection were explained and individuals were informed that they could leave the study at any time. Only those who signed the Informed Consent were included in the sample. All protocols were reviewed and approved by the Research Ethics Committee of the *Universidade Estadual Paulista* (case number 26/2009).

### Body composition

For body composition analysis we used the 3-compartment model dual energy X-ray absorptiometry (DXA), Lunar brand°, model DPX-MD, software 4.7 equipment. This technique allows to estimate whole and segmented body composition (lean mass, fat mass and body mineral density of trunk, upper and lower limbs). Data were transmitted to a device connected to a computer on which results of lean mass, body fat and bone mineral density were recorded.

### Gait speed

A 3-meter walking test was used to assess the gait speed. Subjects were instructed to walk naturally, and the lower time (in seconds) obtained between two walks was recorded.

### Definition of groups

The sample was divided into four groups: Normal Group (NG), with subjects who were not obese or sarcopenic; Obesity Group (OG), with subjects who had a fat percentage above the 60th percentile (34.1 and 44.2%, for men and women, respectively), according to the recommendations of Baumgartner et al.,^(^ ^14^ ^)^ Sarcopenia Group (SG), with subjects with low muscle mass and low gait speed were classified as sarcopenic; and Sarcopenic Obesity Group (SOG), with subjects who had both these unfavorable conditions (obesity and sarcopenia).

For muscle mass classification, the appendicular lean mass (ALM) index was used (upper limb + lower limb lean mass [kg]/stature [m]^2^), the individuals with ALM index below 7.59kg/m^2^ and 5.57kg/m^2^ for men and women, respectively, were considered with low muscle mass. The adoption of these cutoff points was based on two standard deviations below the mean of a reference group of Brazilian young adults (n=60; 25 men and 35 women) aged between 20 and 30 years.^(^ ^15^ ^)^ Subjects with gait speed below 0.8m/s in the 3-meters walking test, were considered with low gait speed.^(^ ^3^ ^)^

### Mobility

Mobility was defined by performance achieved in static balance test; and sit-to-stand test, from the Short Physical Performance modified Battery.^(^ ^16^ ^)^

The static balance test has four steps, performed in a sequence (10 seconds each): to stand with feet together, side by side; to touch the heel of one foot on the side of the big toe of the opposite foot, semi-tandem; to balance on one foot, first with either foot and then with the other; and to stand with one foot in front of the other.

Each measurement was considered successful when the individual could remain for 10 seconds in the mentioned position. The possible scores for this test were: zero if disability, unable to perform any action for the stipulated time; 1 if individual could hold a side-by-side stand position but was unable to hold a semi-tandem stand position; 2 if individual could hold a semi-tandem stand position but was unable to stand on one foot; 3 if individual could stand on one foot but was unable to hold a tandem stand position; 4 if individual could held the full tandem stand position.

To measure the strength of the lower limbs the sit-to-stand test was applied, in which subjects kept their arms crossed over chest, and, at a signal from the evaluator, stood up and sat down in the chair as quickly as possible, five times without pause. Those who failed to perform this task in less than 60 seconds were disqualified from the test. Scores attributed to this test were: zero if not able to perform the test; 1 if time greater than or equal to 16.70 seconds; 2 if time taken between 13.70 and 16.69 seconds; 3 if time taken between 11.20 and 13.69 seconds; 4 if time taken less than or equal to 11.19 seconds.

For mobility classification we considered performance by sum of two tests (zero to 8 points), with those with a total score below 25 percentile (3 points), whom were considered as lower mobility individuals.

### Osteoarticular diseases

#### Osteopenia and osteoporosis

We used the DXA to identify osteopenia and osteoporosis. Bone mineral density of the total proximal femur was analyzed based on manufacturer protocol by an experienced technician. Individuals were classified as osteopenia or osteoporosis according to the criteria established by the World Health Organization.^(^ ^17^ ^)^

### Other osteoarticular diseases

The prevalence of arthritis, osteoarthritis, herniated disc, back pain and scoliosis in the study population was verified by the reported morbidities questionnaire, taken from the Standard Health Questionnaire for Washington State.^(^ ^18^ ^)^ This is a closed survey to identify the presence/absence of chronic diseases, distributed into three groups: cardiovascular, metabolic, and osteoarticular.

### Statistical analysis

The χ^2^ test was used to analyze the proportion of older people in groups (NG, OG, SG and SOG) according to sex as well as to indicate associations between the dependent (mobility) and independent (sarcopenia, obesity and sarcopenic obesity) variables. The independent variables with p<0.20 in the χ^2^ test were included in the multivariate model built by binary logistic regression analysis, adjusted for the control variables (sex and osteoarticular diseases), which expresses the magnitude of associations in values of odds ratio (OR) and their 95% confidence intervals. One-way analysis of variance (ANOVA) was used to compare the mobility of the four groups analyzed followed by the post-hoc Tukey test. Statistical analysis was performed using Statistical Package of Social Science (SPSS) software, version 17.0, and significance level was set at 5%.

## RESULTS

The sample of study consisted of 116 older individuals aged between 80 and 95 years, mean 83.3 (2.7) years. Of these, 69 (60%) were women, mean 83.8 (2.9) years, and 47 (40%) were men, mean 83.3 (2.5) years.

There was no significant difference between groups (NG, OG, SG and SOG) in proportion for osteoarticular diseases (p=0.748).

**Figure 1:**
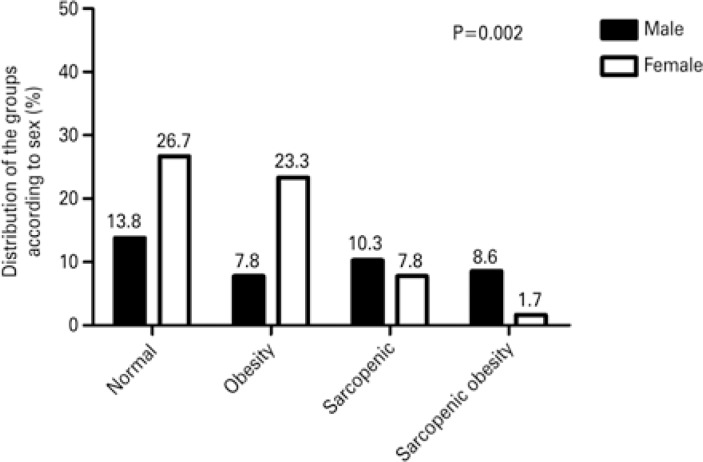
Frequency distribution of the Normal, Obesity, Sarcopenia and Sarcopenic Obesity Groups according to sex

Figure 1 shows the distribution of the sample according to obesity, sarcopenia and sarcopenic obesity and comparison analysis between sexes. Men had higher proportion of sarcopenic obesity (8.6%) and women had a higher proportion of obesity (23.3%).

The comparison of mobility between the four groups is shown in table 1 . The SG group had lower performance in lower limbs strength test (p=0.003) and in sum of two tests (p=0.049) compared with OG and NG groups.

**Table 1:** Comparison of mobility between the Normal, Obesity, Sarcopenia and Sarcopenic Obesity Groups

Tests	Normal (n=47)	Obesity (n=36)	Sarcopenia (n=21)	Sarcopenic obesity (n=12)	f	p value
	Mean (SD)	Mean (SD)	Mean (SD)	Mean (SD)		
Balance	3.0 (1.2)	2.9 (1.3)	2.6 (1.6)	2.7 (1.6)	0.420	0.739
Strength	2.0 (1.4) *	1.9 (1.2) *	0.9 (1.3) †	1.1 (0.9) *†	5.030	0.003
Two tests	5.0 (2.1) *	4.8 (2.1) *	3.6 (2.5) †	3.8 (2.2) *†	2.705	0.049

*† Different letters mean difference between groups

SD: standard deviation

The association between obesity, sarcopenia and sarcopenic obesity and mobility is shown in table 2 . There was association between sarcopenia and reduced mobility.

**Table 2:** Association between obesity, sarcopenia, sarcopenic obesity and mobility of older individuals aged 80 years and older

Groups		Reduced mobility		p value
		Yes n (%)	No n (%)	
Obesity	Yes	5 (13.9)	31 (86.1)	0.627
	No	14 (17.5)	66 (82.5)	
Sarcopenia	Yes	7 (33.3)	14 (66.7)	0.020
	No	12 (12.6)	83 (87.4)	
Sarcopenic obesity	Yes	3 (25.0)	9 (75.0)	0.394
	No	16 (15.4)	88 (84.6)	

In table 3 includes the multiple analysis between sarcopenia and reduced mobility. Older people with sarcopenia had 3.44 times more chances of reduced mobility regardless of sex and presence of osteoarticular diseases, compared with those without sarcopenia.

**Table 3:** Multiple analysis between sarcopenia and reduced mobility of older individuals aged 80 years and older

Sarcopenia	Reduced mobility	p value
	OR * (95%CI95)	
Yes	3.44 (1.12-10.52)	0.031
No	1.00	

* Model adjustment by sex and osteoarticuar diseases. Hosmer and Lemeshow test 0.84.

OR: odds ratio; 95%CI: 95% confidence interval.

## DISCUSSION

This study determined which abnormality of body composition (obesity, sarcopenia or sarcopenic obesity) is related with reduced mobility of Brazilian older people aged 80 years and over. It was observed that older people with sarcopenia had more limited mobility compared with NG, OG, or SOG.

Reduction in lean mass is one of the most frequently used variable for indicative losses in mobility.^(^ ^7^ ^)^ Men have larger amounts of lean mass and a higher incidence of sarcopenia compared to women.^(^ ^19^ ^)^ Our findings corroborate with previously studies in which the proportion of older people aged 80 years and older with sarcopenia was higher in men (10.3%) than in women (7.8%) and, consequently, sarcopenic obesity − 8.6% and 1.7%, respectively.

Sarcopenia is related to mobility and functional impairment in older people older than 60 years.^(^ ^20^ ^)^ Also sarcopenia influences mobility, however, the amount of fat and body size should also be considered for such analysis.^(^ ^6^ ^)^ In our study, sarcopenia alone was associated with reduced the mobility, and increased the chances of an older people aged 80 years and older to present reduced mobility by 3.44 times, regardless of the sex and presence of osteoarticular diseases.

Women had higher proportions of body fat.^(^ ^21^ ^)^ Gomes et al.,^(^ ^22^ ^)^ also observed this evidence in women aged 80 years and older, similar to results found in this study, in which 23.3% of the women were identified with obesity compared with 7.8% of men.

A higher quantity of fat mass or higher proportion of body fat can increase the body overload, limit movements and impose additional stress on joints and muscles, thereby accentuating the risk of disability.^(^ ^23^ ^)^ In our study, obesity was not a limiting factor for mobility. Similar to our results, Sallinen et al.,^(^ ^24^ ^)^ also found no association between body fat and mobility in older people aged 80 years and older. These results may indicate that body fat does not interfere in the mobility of the elderly aged 80 years and older, since it tends to redistribute and reduce with aging.^(^ ^25^ ^)^

Sarcopenic obesity represents a challenge for health professionals who need to apply appropriate interventions in elderly population in order to reduce the risk that excess fat can cause to their health whilst preserving lean mass.^(^ ^26^ ^)^ Due to these factors, sarcopenic obesity is considered as one of the most damaging morphological conditions to both mobility and the general health of older people.^(^ ^27^ ^)^

This association was observed by Stenholm et al.,^(^ ^11^ ^)^ who found that sarcopenic obesity increased the risk of reduction in walking speed and mobility impairments in the elderly over 65 years. Our findings differ from those found in the study mentioned because older people aged 80 years and older with sarcopenic obesity showed no low mobility compared with other groups.

Level of physical activity affected the components of lean mass and body fat. Over time, the elderly tend to become increasingly sedentary which leads to a vicious cycle of reduced mobility, and with the reduction in mobility the levels of physical activity become even more low.^(^ ^28^ ^)^ Carmo et al.,^(^ ^29^ ^)^ demonstrated the influence of physical activity when they showed that mobility for activities such as walking, sitting and rising from a chair and getting up it from the prone position was more preserved in elderly women who were physically active. Therefore, physical activity should be viewed as a solution to the loss of mobility by preserving physical^(^ ^29^ ^)^ and body^(^ ^30^ ^)^ components.

Despite the relevance of the results found in our study, some limitations were: no use of control variables as physical activity, energy intake and socioeconomic status, analogue scale of pain; no use of ADL questionnaires; the cross-sectional design of the study limited the ability of establishes causal relationships. However, it must be mention that studies to verify such aspects in older people aged 80 years and older are still scarce.

## CONCLUSION

Older people aged 80 years and older with sarcopenia have more chance for reduction in mobility. Preventive measures such as the practice of physical activity, especially in the course of life, can avoid the occurrence of sarcopenia and attenuate mobility reduction in the older people. Further longitudinal surveys are need to observe the causal relationships.
